# Genetically supported protein signals and ferroptosis-related cell states in human intervertebral disc degeneration: local pQTL–FinnGen screening integrated with nucleus pulposus single-cell RNA sequencing

**DOI:** 10.3389/fcell.2026.1944218

**Published:** 2026-10-21

**Authors:** Guangying Tang, Xinjun Wu, Yongchun Lin, Zhiyu Tang, He Liang

**Affiliations:** 1 Department of Bone Injury, Qiannan Buyei and Miao Autonomous Prefecture Hospital of Traditional Chinese Medicine, Duyun, Guizhou, China; 2 Department of Orthopedics, The Second People’s Hospital of Kashgar Prefecture, Kashgar, Xinjiang, China; 3 Outpatient Department Physical Examination Center, General Hospital of Western Theater of Chinese People’s Liberation Army, Chengdu, Sichuan, China; 4 Department of Orthopaedics, Songjiang Hospital Affiliated to Shanghai Jiao Tong University School of Medicine, Shanghai, China

**Keywords:** ferroptosis, FinnGen, intervertebral disc degeneration, Mendelian randomization, nucleus pulposus, protein quantitative trait locus, single-cell RNA sequencing, TFRC

## Abstract

**Background:**

Intervertebral disc degeneration (IVDD) involves nucleus pulposus (NP) cell dysfunction, inflammatory remodeling, and oxidative stress. Genetic support for circulating protein signals and their cell-type-specific context remains incompletely defined.

**Methods:**

We integrated deCODE cis-pQTL data with FinnGen R12 M13_INTERVERTEB and human NP scRNA-seq (GSE153066). Local MR screening used harmonized variants with Benjamini–Hochberg correction. The scRNA-seq analysis retained 70,622 cells from 16 libraries and used library-level pseudobulk comparisons. A qPCR validation experiment in PMA-differentiated THP-1 cells (ATCC TIB-202) evaluated MST1, SAA1, BPI, and GCLM under an inflammatory condition.

**Results:**

Four multi-SNP signals passed correction: MST1 showed higher endpoint odds (OR = 1.057, q = 4.15 × 10−6), whereas DLK1 (OR = 0.933, q = 0.016), ITIH1 (OR = 0.926, q = 0.016), and SAA1 (OR = 0.927, q = 0.023) showed inverse associations. TP53I3 and BPI remained nominal exploratory signals. TFRC showed a BH-positive single-SNP result (OR = 1.471, q = 0.023). ScRNA-seq resolved eight broad cell populations and demonstrated between-library heterogeneity; nominal macrophage-composition and selected expression differences did not survive within-panel correction. In the qPCR experiment, relative expression was 1.34 for MST1, 0.31 for SAA1, 3.46 for BPI, and 1.70 for GCLM (control = 1.0; n = 4 independent biological replicates/group; all Welch P < 0.001), consistent with the scRNA-seq directions.

**Conclusions:**

The study identifies genetically supported protein signals and ferroptosis-related cellular expression patterns while separating genetic screening from transcriptomic localization. The qPCR experiment provides orthogonal experimental support for selected candidate expression changes in the THP-1 inflammatory model.

## 1 Introduction

Intervertebral disc degeneration (IVDD) is a common age-related spinal pathology and an important structural contributor to degenerative spine disorders and low back pain. Low back pain remains the leading cause of years lived with disability worldwide, while longitudinal imaging studies demonstrate that disc degeneration is frequent and progresses with age (GBD 2021 Low Back Pain Collaborators, 2023; Teraguchi et al., 2017). IVDD should nevertheless be distinguished from low back pain as a clinical syndrome, because imaging-defined degeneration is common even in individuals without symptoms.

At the tissue level, degeneration involves loss of nucleus pulposus (NP) cell homeostasis, extracellular matrix (ECM) breakdown, inflammatory signaling, and altered mechanical function. Cytokine-driven catabolism and matrix-remodeling enzymes contribute to progressive deterioration of disc structure (Risbud and Shapiro, 2014; Zou et al., 2023). Oxidative stress is also increasingly recognized as a major component of the degenerative microenvironment and can influence cellular senescence, death, and matrix metabolism (Wang et al., 2023).

Ferroptosis is an iron-dependent form of regulated cell death driven by lipid peroxidation (Dixon et al., 2012). Experimental studies in NP cells and animal models have linked disturbed iron handling and ferroptotic signaling to disc degeneration, including ferroportin-dependent iron homeostasis and HMOX1/GPX4-related mechanisms (Lu et al., 2021; Yao et al., 2023). Single-cell analysis of human NP tissue has further identified ferroptosis-associated transcriptional patterns in discrete chondrocyte-like cell states (Zhang et al., 2021). These observations support examining ferroptosis-related genes in parallel with broader disease-associated protein signals rather than assuming that all cell-death programs are uniformly activated across the disc.

Single-cell RNA sequencing (scRNA-seq) provides a complementary layer of resolution by separating transcriptional programs across heterogeneous NP, stromal, and immune populations. Human disc scRNA-seq studies have reported distinct NP cell states and highlighted macrophage-associated inflammatory interactions during degeneration (Zhang et al., 2021; Ling et al., 2022; Han et al., 2022). However, cell-level visualization alone does not provide patient-level inference, and analyses of public datasets require careful attention to biological replication and library-level variation.

Protein quantitative trait locus (pQTL) resources offer a genetic route for prioritizing circulating proteins associated with disease outcomes. Large-scale plasma proteogenomic studies have mapped thousands of pQTLs (Ferkingstad et al., 2021), while Mendelian randomization (MR) can use genetic variants as instrumental variables to test exposure-outcome relationships under explicit assumptions (Lawlor et al., 2008). Reporting guidance emphasizes instrument validity, linkage disequilibrium (LD), sensitivity analyses, and cautious causal interpretation when these assumptions cannot be fully evaluated (Skrivankova et al., 2021). Transferrin receptor 1 (TFRC), a central iron-uptake protein, has also been experimentally validated as a ferroptosis marker (Feng et al., 2020).

Accordingly, the present study integrates a local deCODE cis-pQTL-FinnGen screen with library-aware analysis of the human NP scRNA-seq dataset GSE153066. The primary aim was to identify FDR-supported genetic screening signals, place selected genes within the cellular architecture of degenerated NP tissue, and examine a prespecified ferroptosis-related gene panel while keeping genetic evidence, cell localization, and library-level expression effects analytically distinct.

## 2 Materials and methods

### 2.1 Data sources and study contract

The study question, disease, organ, specimen, comparison, statistical units, and eight-figure map were fixed before figure construction. The genetic exposure was the supplied deCODE cis-pQTL summary table. The outcome was FinnGen R12 M13_INTERVERTEB, labelled in the release as other intervertebral disc disorders. The single-cell dataset was GEO GSE153066, titled Single cell sequencing of human nucleus pulposus, generated on GPL24676 (Illumina NovaSeq 6000). Official sample metadata identify eight relatively normal libraries (CTL1–CTL8; GSM4633740–GSM4633747) and eight degenerated libraries (IDD1–IDD8; GSM4633748–GSM4633755). Figure 1 was added subsequently as an experimental qPCR validation module and was not part of the original eight-figure analytical plan.

**FIGURE 1:**
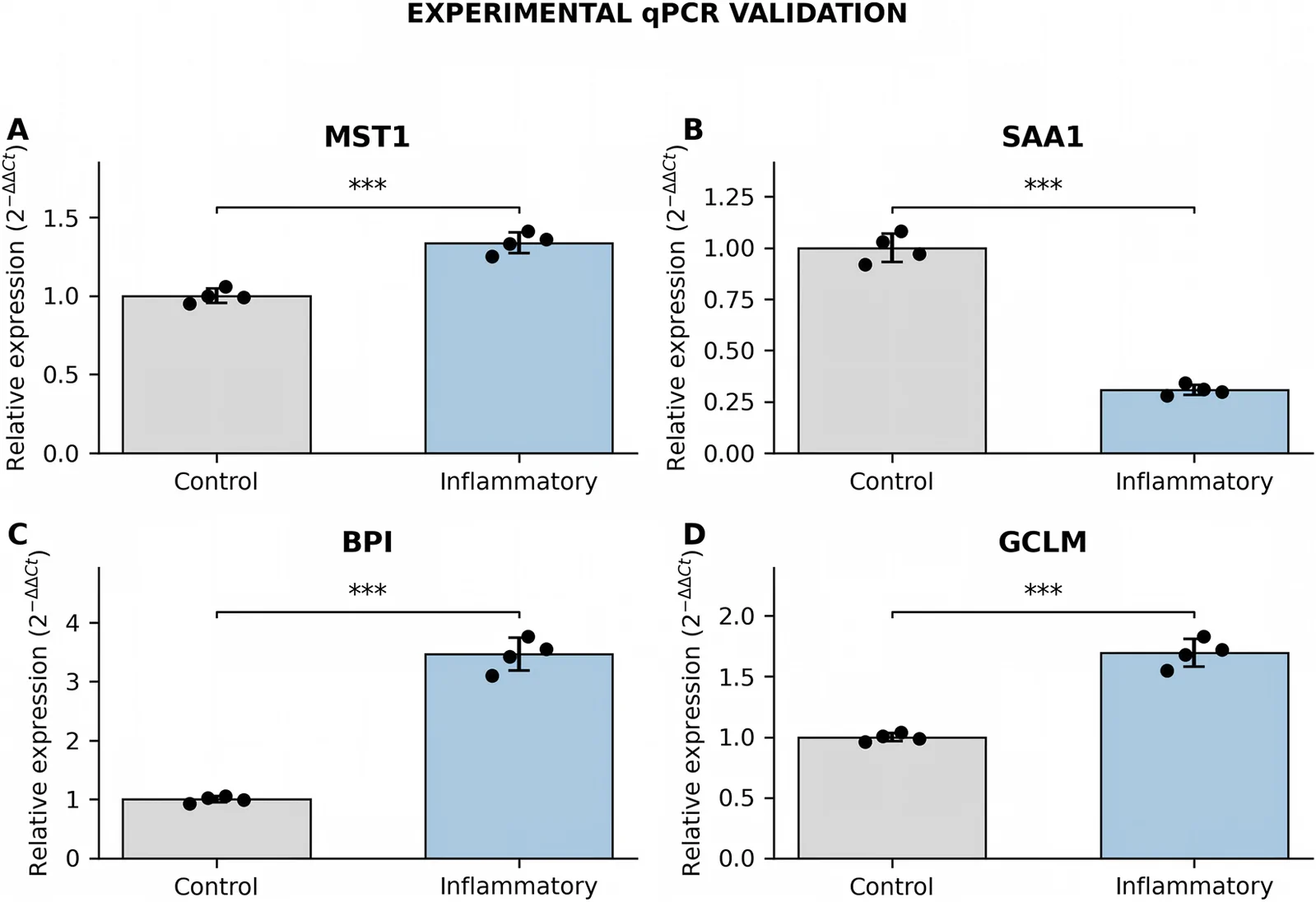
Experimental qPCR validation of four representative follow-up genes in an ATCC macrophage-like cell model. THP-1 cells (ATCC TIB-202) were used as the experimental model. **(A)**, MST1; **(B)**, SAA1; **(C)**, BPI; **(D)**, GCLM. Bars show mean ± SD of four independent biological replicates, and dots show the individual replicate values. Control was normalized to 1.0 using the (2^−ΔΔCt^) framework. Under the inflammatory condition, relative expression was 1.34 ± 0.07 for MST1, 0.31 ± 0.03 for SAA1, 3.46 ± 0.28 for BPI, and 1.70 ± 0.12 for GCLM. Two-sided Welch t tests were used; ***P < 0.001 for each comparison.

### 2.2 Local pQTL–FinnGen screening

Exposure variants were restricted to the genome-wide significant rows supplied in the pQTL table. Alleles were harmonized to the FinnGen outcome effect allele; mid-frequency palindromic variants with unresolved orientation were excluded. We calculated single-SNP Wald ratios, gene-level IVW random-effects estimates when multiple usable SNPs were available, weighted-median estimates, MR-Egger slopes, heterogeneity statistics, Egger intercepts, and leave-one-out estimates. Benjamini–Hochberg correction was applied across the 1,723 gene-level primary tests. The six-gene fixed figure set was defined before plotting: four q < 0.05 multi-SNP screening genes and two directionally concordant nominal exploratory genes that were present in the single-cell matrix.

The supplied exposure table does not include a reference LD panel or an auditable clumping record. Consequently, the multi-SNP estimates are reported as local MR screening signals, not confirmed independent-instrument causal effects. The same boundary applies to the funnel, Egger, and leave-one-out displays.

### 2.3 Single-cell preprocessing and annotation

The GSE153066 matrix was read as a genes-by-cell raw count matrix with 36,847 genes and 85,205 barcode columns. QC followed the source-study thresholds: detected genes >200 and <5,000, and mitochondrial counts <20%. This retained 70,622 cells. Counts were normalized to 10,000 total counts per cell and log1p transformed for cell-level display. Highly variable genes, PCA, neighbors, UMAP, Leiden clustering, and marker ranking were calculated in the current environment. Broad labels were assigned from marker overlap and cluster marker ranking; low-specificity clusters were retained as Stress_unresolved rather than forced into a canonical lineage. The label set used in the figures was B_cell, Erythroid_like, Fibroblast_AF, Macrophage, NK_cell, NP_chondrocyte, Stress_unresolved, T_cell.

### 2.4 Library-level summaries and ferroptosis panel

Condition comparisons were anchored to the 16 GEO libraries (8 CTL versus 8 IDD), not to individual cells. Raw counts were summed within each library and converted to log2(CPM+1) for pseudobulk effects. Cell-level UMAPs, marker displays, and feature maps are descriptive. A six-gene ferroptosis-related pQTL panel (TFRC, FTL, AKR1C3, GCLM, HMOX1, HSPB1) was carried forward independently of MR significance, and a transparent marker program additionally included GPX4, SLC7A11, ACSL4, FTH1, NFE2L2, and PTGS2. This is a prespecified follow-up panel, not a claim of a complete FerrDb catalogue.

### 2.5 Statistical reporting

For library-level comparisons, Welch tests and 95% descriptive intervals were used where shown, with Benjamini–Hochberg q values retained in the result tables. The CTL and IDD libraries are unpaired, and the GEO age distributions are imbalanced (CTL median 34.5 years versus IDD median 43 years). No age-adjusted or paired estimate was substituted for the unpaired screen. Positive log2FC and percentage-point effects throughout the single-cell results mean IDD minus CTL.

### 2.6 *In vitro* qPCR validation

To provide orthogonal wet-laboratory validation of selected candidates from the computational workflow, qPCR experiments were performed in THP-1 cells (ATCC TIB-202), a human monocytic cell line that can be differentiated into macrophage-like cells (Tsuchiya et al., 1980). Cells were cultured in RPMI-1640 with 10% fetal bovine serum and 0.05 mM 2-mercaptoethanol, differentiated with phorbol 12-myristate 13-acetate (PMA; 100 nM, 24 h) followed by a 24-h recovery, and then exposed to IL-1β and TNF-α (10 ng/mL each, 24 h) as a degenerative inflammatory condition. Four genes were selected directly from the existing analyses: MST1 and SAA1 as FDR-positive multi-SNP screening signals, BPI as the largest positive follow-up pseudobulk effect, and GCLM as a positive ferroptosis-panel expression effect. Ct-derived relative expression values were normalized to GAPDH and calculated using the (2^−ΔΔCt^) method (Livak and Schmittgen, 2001). Four independent biological replicates per group were analyzed. Two-sided Welch t tests were used for between-group comparisons, with P < 0.05 considered nominally significant. Because PMA exposure and post-differentiation recovery conditions can influence THP-1 macrophage phenotype and inflammatory responsiveness (Daigneault et al., 2010; Lund et al., 2016), these conditions are reported explicitly; THP-1 is also widely used as an *in vitro* monocyte/macrophage model (Chanput et al., 2014).

## 3 Results

### 3.1 Genetic screening defines a cautious six-gene follow-up set

After harmonization, 5,820 usable SNP rows supported gene-level screening across 1,723 genes. The four fixed multi-SNP q < 0.05 signals were MST1 (OR 1.057), DLK1 (OR 0.933), ITIH1 (OR 0.926), and SAA1 (OR 0.927); the complete six-gene results are summarized in Table 1. Figure 2A shows the MST1 method-level estimates and their confidence intervals, and Figure 2B shows the corresponding DLK1 comparison.

**TABLE 1:** Fixed six-gene local MR screening set. ORs are for FinnGen R12 M13_INTERVERTEB and are not presented as confirmed causal protein effects.

Gene	SNPs	OR	95% CI	P	BH q	Tier
MST1	12	1.057	1.038–1.077	2.41e-09	4.15e-06	FDR<0.05
DLK1	6	0.933	0.903–0.964	2.84e-05	0.016	FDR<0.05
ITIH1	8	0.926	0.893–0.961	4.76e-05	0.016	FDR<0.05
SAA1	6	0.927	0.892–0.962	8.07e-05	0.023	FDR<0.05
TP53I3	6	0.952	0.923–0.982	0.0017	0.13	Nominal only
BPI	5	0.949	0.916–0.983	0.0035	0.17	Nominal only

**FIGURE 2:**
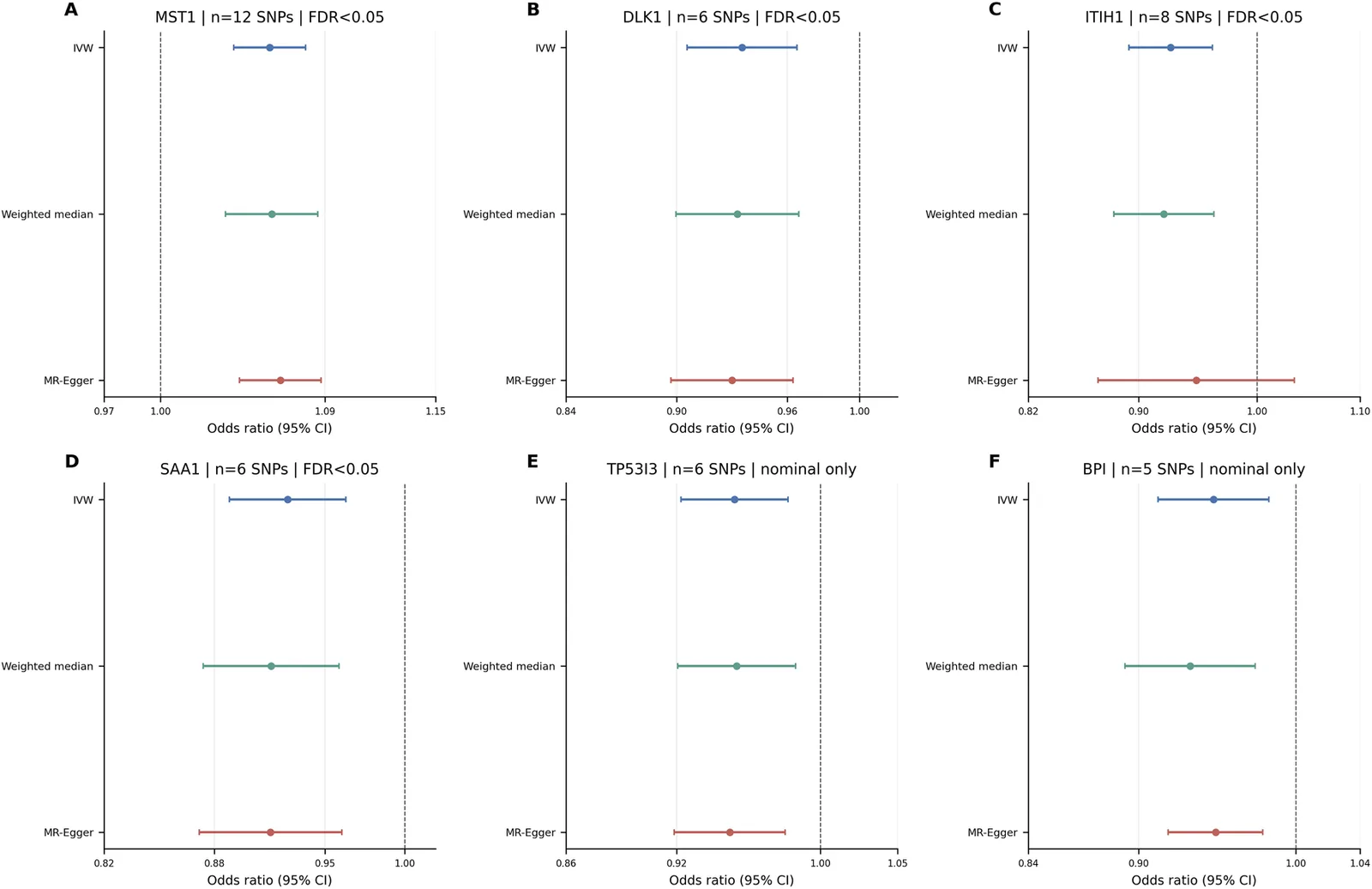
Local MR method comparison for the fixed six-gene set. **(A)**, MST1; IVW, weighted-median, and MR-Egger odds ratios with 95% intervals from 12 harmonized SNPs. **(B)**, DLK1; six SNPs and the same three method summaries. **(C)**, ITIH1; eight SNPs and method-level intervals. **(D)**, SAA1; six SNPs and method-level intervals. **(E)**, TP53I3; six SNPs, retained as nominal exploratory evidence because BH q exceeds 0.05. **(F)**, BPI; five SNPs, also nominal exploratory evidence. The dashed vertical line is OR = 1. The direction of association (OR above or below 1) is inherited from the harmonized FinnGen effect allele; panels are local screening displays, not confirmed causal estimates.

The same method comparison is shown for ITIH1 in Figure 2C and SAA1 in Figure 2D. The weighted-median and MR-Egger directions are concordant with IVW for these four q-positive genes, but their intervals are not a substitute for LD validation. Figures 2E,F retain TP53I3 and BPI as nominal exploratory members of the fixed display set; their q values are above 0.05 and they are not described as FDR discoveries.

At the SNP level, the exposure–outcome relationship for MST1 is shown in Figure 3A and for DLK1 in Figure 3B. These panels show harmonized beta pairs and inherited method lines; they do not add a new significance test. The corresponding scatter displays for ITIH1 and SAA1 are shown in Figures 3C,D Figures 3E,F retain TP53I3 and BPI as exploratory comparators.

**FIGURE 3:**
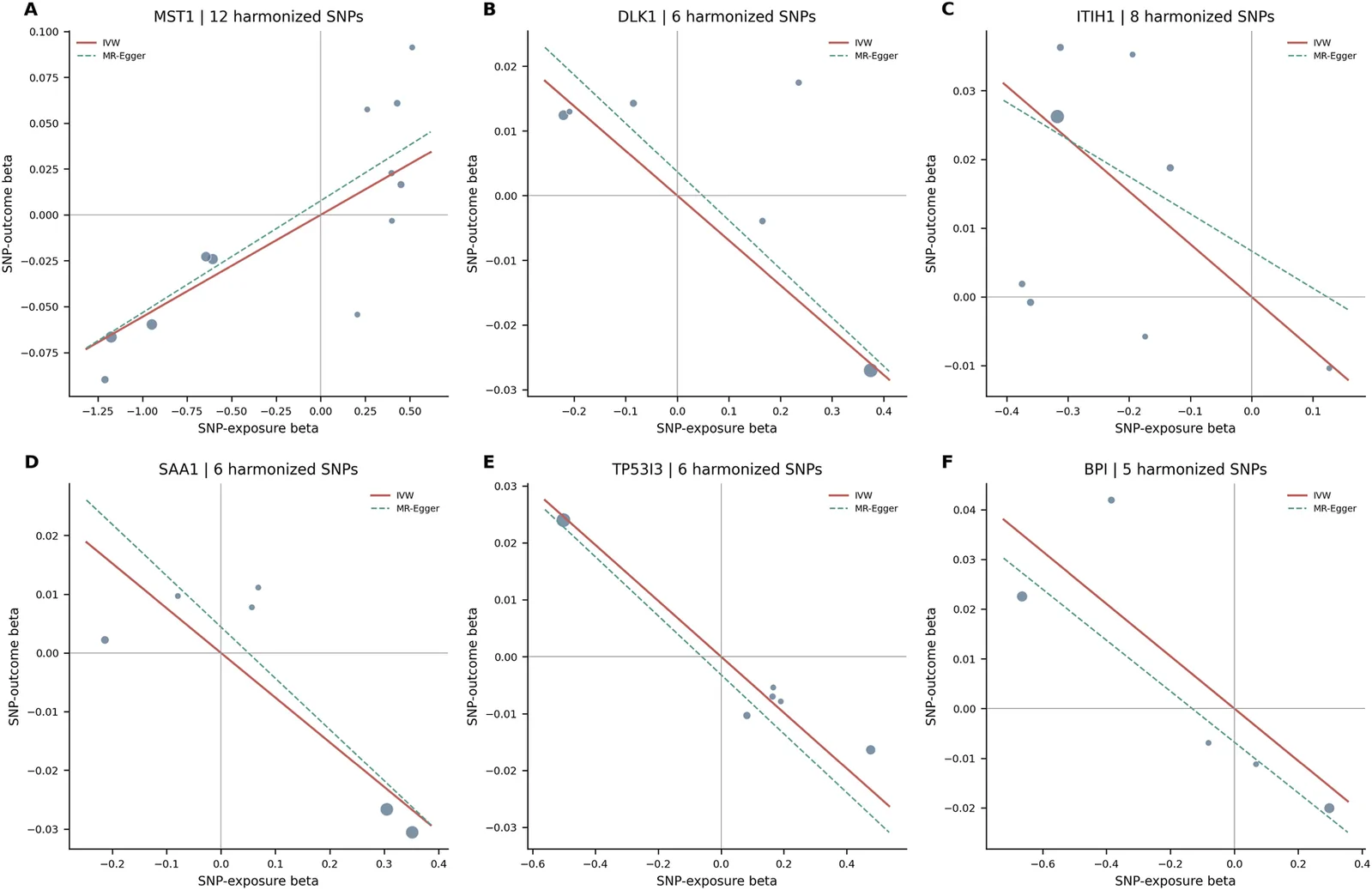
SNP exposure–outcome scatter plots for the fixed six-gene set. **(A)**, MST1; **(B)**, DLK1; **(C)**, ITIH1; **(D)**, SAA1; **(E)**, TP53I3; **(F)**, BPI. Each point is a harmonized SNP, point size scales with the supplied Wald precision, the solid line is the gene-level IVW estimate, and the dashed line is the MR-Egger slope with intercept when available. Axes are SNP-exposure beta and harmonized SNP-outcome beta. No new significance test is performed in this display.

Precision-based single-SNP displays for MST1 and DLK1 are shown in Figures 4A,B. The points are plotted against 1/SE so that estimate dispersion is visible without treating cell or SNP counts as independent patients. Figures 4C,D show the same precision display for ITIH1 and SAA1. Figures 4E,F retain TP53I3 and BPI; apparent asymmetry is not interpreted as proof of pleiotropy.

**FIGURE 4:**
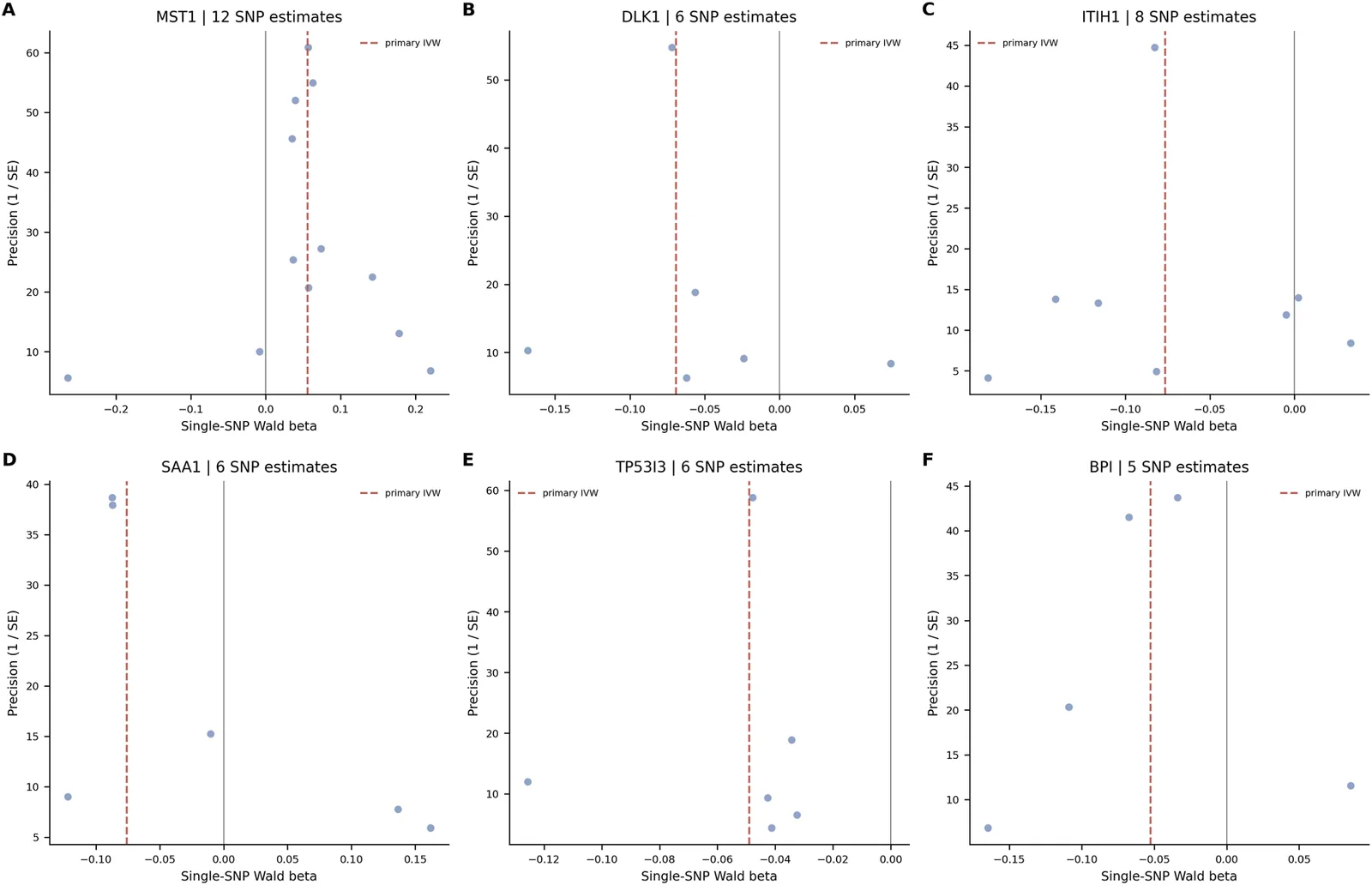
Single-SNP Wald-ratio precision displays. **A–F**, genes in the fixed order MST1, DLK1, ITIH1, SAA1, TP53I3, and BPI. Each point is a single-SNP Wald beta plotted against 1/SE; the dashed line is the primary gene-level IVW beta and the solid reference line is beta = 0. The displays describe estimate dispersion and do not assert funnel symmetry or absence of pleiotropy.

Leave-one-out estimates for MST1 and DLK1 are shown in Figures 5A,B. Both retain their original direction after each displayed omission. Figures 5C,D show the corresponding sensitivity estimates for ITIH1 and SAA1. MST1 and SAA1 retained nominal significance (P < 0.05) after every displayed omission, while the other genes had at least one omission with P ≥ 0.05; the sign of each estimate was preserved. Figures 5E,F report TP53I3 and BPI without upgrading their exploratory evidence tier.

**FIGURE 5:**
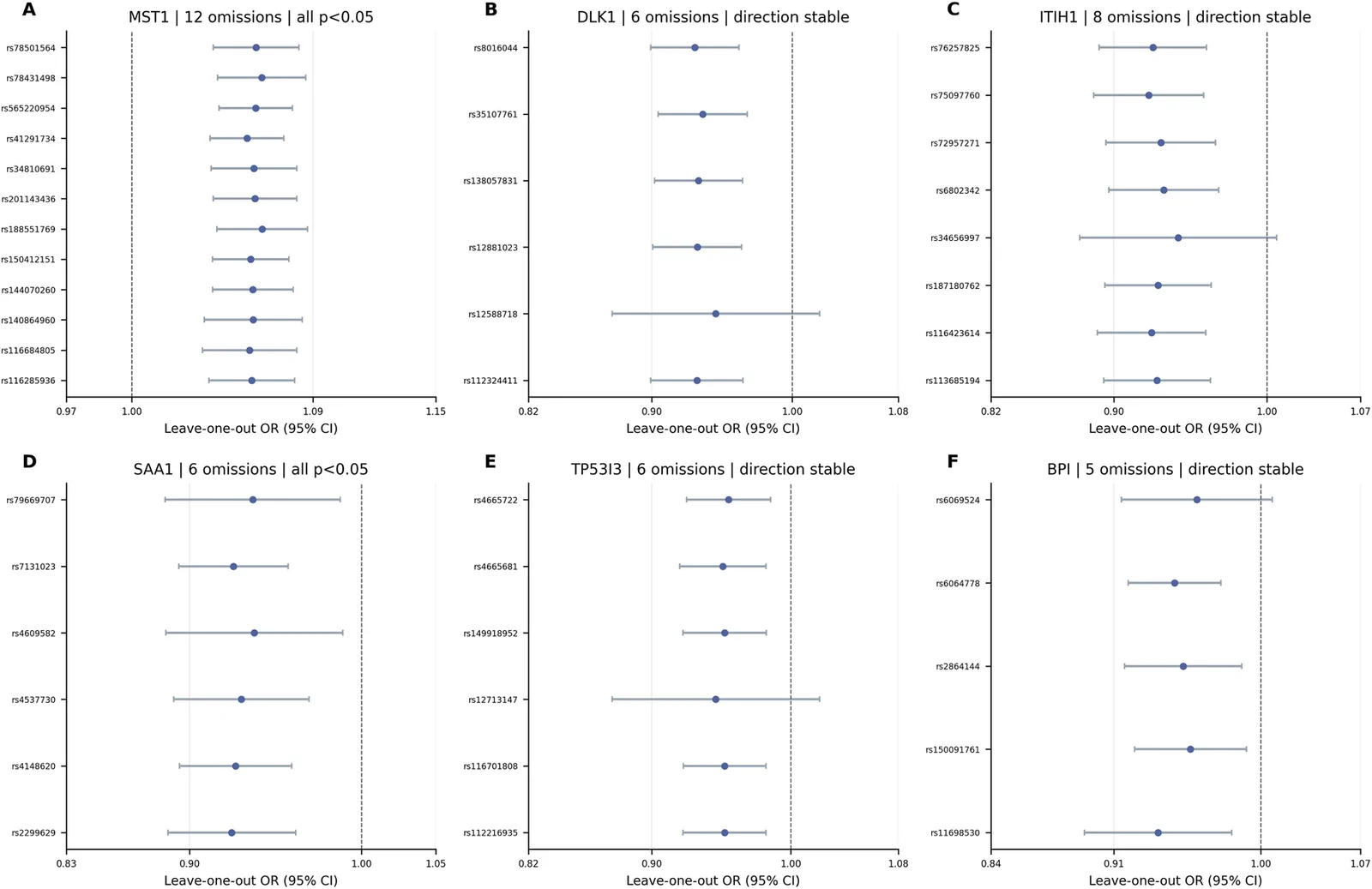
Leave-one-out sensitivity for the fixed six-gene set. **(A)**, MST1; **(B)**, DLK1; **(C)**, ITIH1; **(D)**, SAA1; **(E)**, TP53I3; **(F)**, BPI. Each row omits one harmonized SNP and shows the resulting IVW odds ratio with a 95% interval. MST1 and SAA1 retain P < 0.05 after every omission; the remaining panels retain direction but include at least one nominally non-significant omission. The plot is sensitivity evidence within the supplied instrument set, not LD validation.

### 3.2 GSE153066 provides a library-aware nucleus-pulposus atlas

Figure 6A shows the input and QC-retained barcodes for each of the 16 libraries; the total changed from 85,205 input columns to 70,622 retained cells. Figure 6B displays the library pseudobulk PCA, with CTL and IDD points retained as individual libraries rather than pooled cells. Figure 6C maps the marker-supported labels in UMAP space, and Figure 6D shows the degree of cell-level overlap between CTL and IDD. Both panels are descriptive cell displays. Figure 6E displays reference-marker coherence, including the explicitly unresolved stress cluster and erythroid-like cluster. Figure 6F shows the composition of each library; these percentages are descriptive denominators for the eight-versus-eight library comparisons.

**FIGURE 6:**
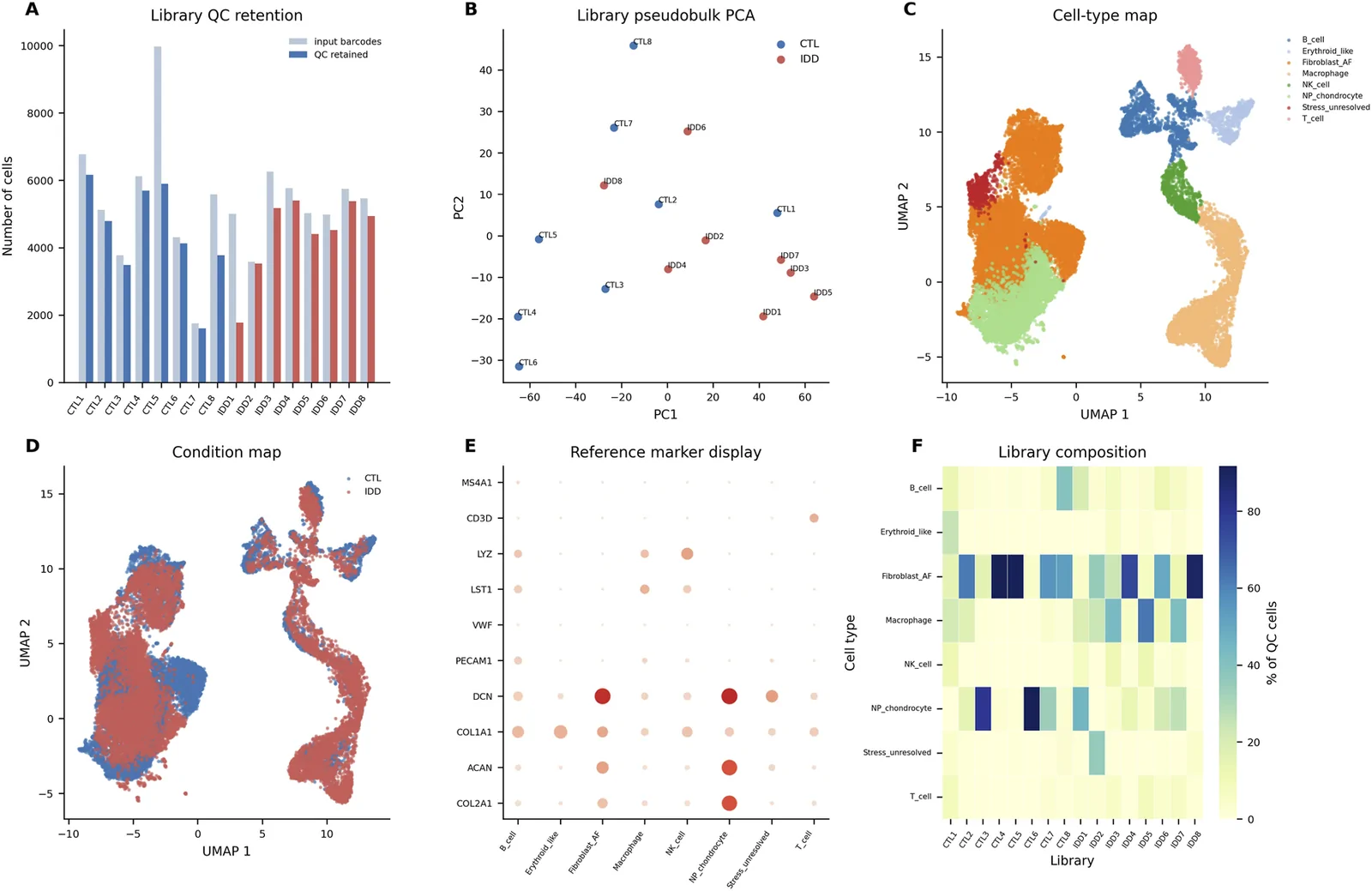
GSE153066 nucleus-pulposus atlas and library-aware QC. **(A)**, input versus QC-retained barcode columns for 16 libraries (85,205 input; 70,622 retained) using detected genes >200 and <5,000 and mitochondrial fraction <20%. **(B)**, PCA of library pseudobulk log2(CPM+1) values using the 500 most variable genes for display; each point is a GSM/library. **(C)**, marker-supported broad cell labels on the UMAP; labels include NP_chondrocyte, Fibroblast_AF, Macrophage, lymphoid, Erythroid_like, and Stress_unresolved. **(D)**, the same UMAP coloured by CTL or IDD condition; this is a descriptive cell map. **(E)**, reference-marker display; point size is percent positive and colour is mean log1p (CP10 K). **(F)**, percent composition of QC-retained cells within each library. Percentages are descriptive and do not represent patient-level inference.

### 3.3 Ferroptosis-related pQTL signals are separated from expression follow-up

The six-gene ferroptosis-related pQTL panel was kept separate from the four q-positive multi-SNP genes. TFRC was the only panel member with a nominal and BH-positive single-SNP screening result (OR 1.471, 95% CI 1.212–1.785, P 9.42e-05, q 0.023). FTL, AKR1C3, GCLM, HMOX1, and HSPB1 were not positive in the genetic screen and therefore enter the manuscript as expression markers. Figure 7A makes this distinction explicit, and Figure 7B shows broad cell-type localization. The TFRC and MST1 feature maps are shown in Figures 7C,D. Figures 7E compares the six-gene ferroptosis-pQTL score across libraries; the Welch P is reported as an exploratory library-level comparison, not a cell-level test. Figure 7F preserves the individual library profiles so that a pooled mean does not conceal between-library heterogeneity.

**FIGURE 7:**
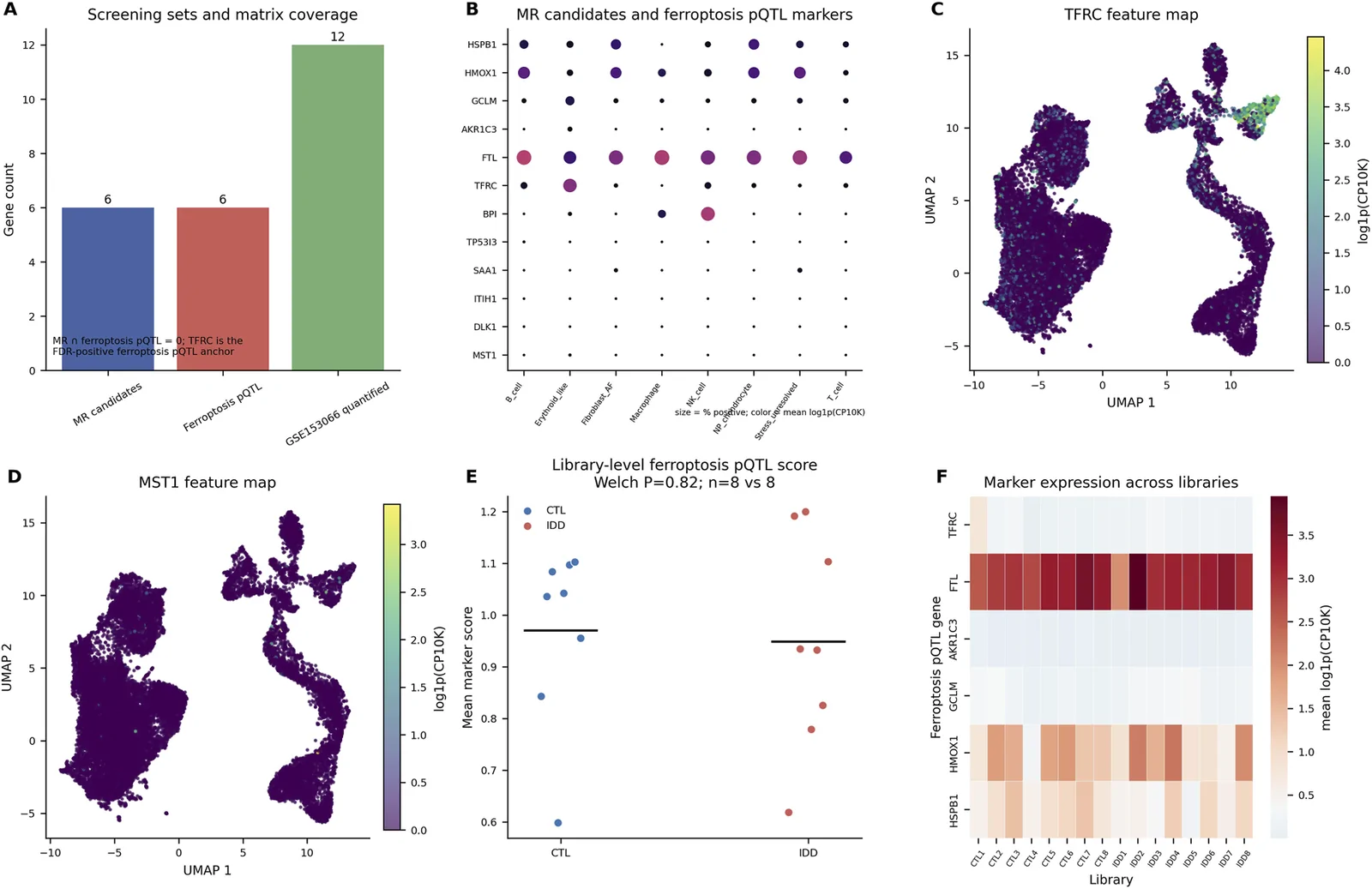
Localization of MR follow-up genes and ferroptosis-related pQTL markers. **(A)**, counts of the six-gene MR follow-up set, six-gene ferroptosis-related pQTL set, and the 12 genes quantified in GSE153066; the sets are not treated as mutually exclusive discoveries. **(B)**, dot display across broad labels for the 12 follow-up genes; size is percent positive and colour is mean log1p (CP10 K). **(C)**, TFRC feature map on the UMAP. **(D)**, MST1 feature map on the UMAP. **(E)**, six-gene ferroptosis-pQTL marker score by library, with Welch P and n = 8 versus 8 shown for orientation only. **(F)**, library-level mean expression for TFRC, FTL, AKR1C3, GCLM, HMOX1, and HSPB1. The panel separates marker localization from the single-SNP TFRC screening result.

### 3.4 Library-level effects connect selected genes to cell-state context

The composition effect panel in Figure 8A places the library-level differences on a common percentage-point scale, while Figure 8B retains the five largest absolute components by the prespecified ranking rule. The macrophage component had the largest positive nominal difference (20.58 percentage points, P 0.031, q 0.250), so it is described as an exploratory pattern rather than an FDR-positive composition result. Figure 8C shows the complete finite-gene library-pseudobulk effect distribution; follow-up labels are overlaid without filtering the cloud to positive genes. In Figure 8D, the cell-type pseudobulk matrix retains the sign of each gene-by-label contrast. Figure 8E summarizes the 12 follow-up genes: BPI had the largest positive library effect among the displayed genes (log2FC 2.932, P 0.035, q 0.27), while GCLM was also positive (log2FC 0.812, P 0.044, q 0.27); neither passes the within-follow-up BH threshold. Figure 8F shows TFRC, MST1, and SAA1 profiles library by library.

**FIGURE 8:**
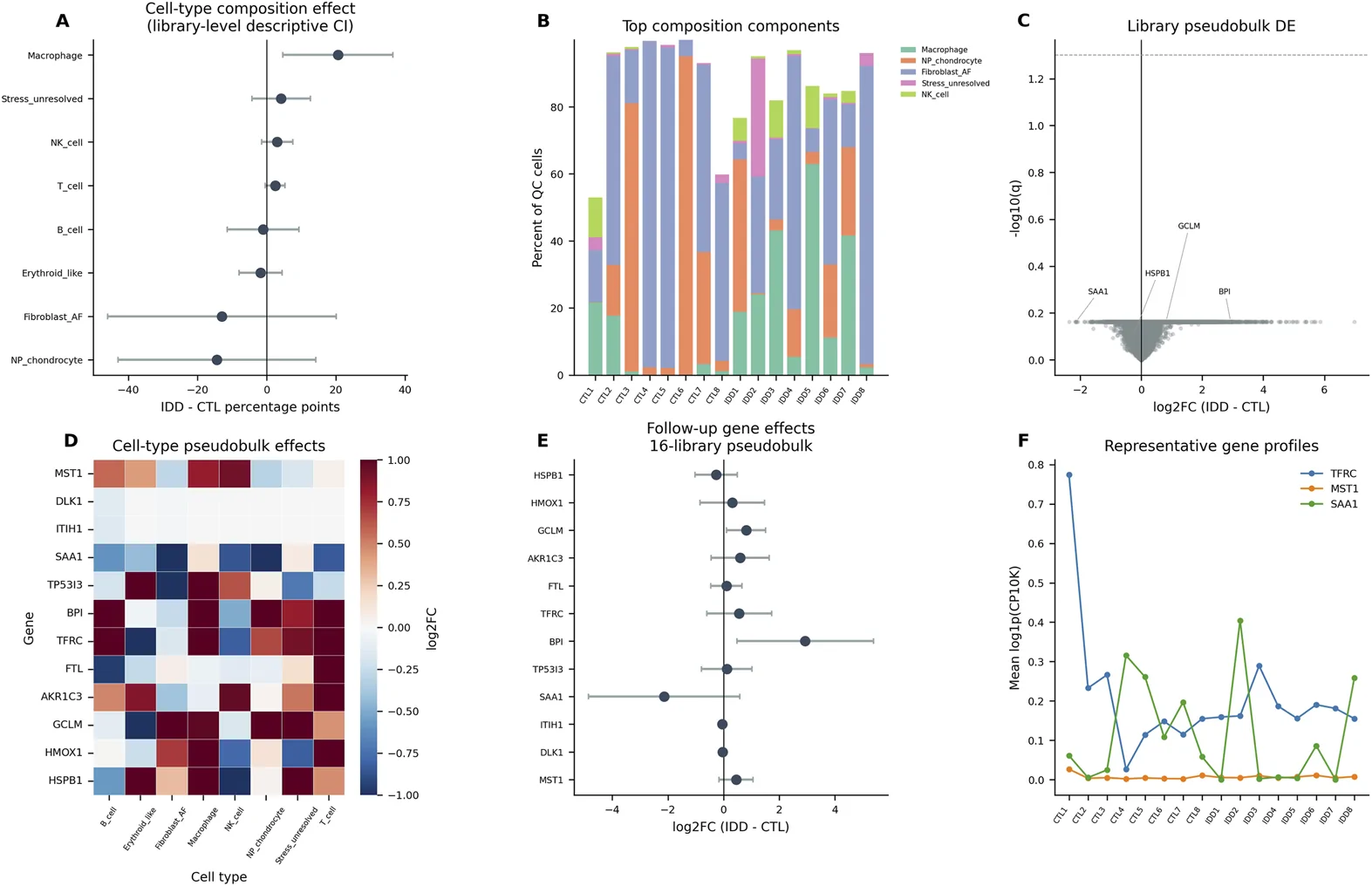
Exploratory IDD-minus-CTL library effects. **(A)**, cell-type composition effects in percentage points with descriptive 95% intervals; positive values mean a larger IDD library fraction. **(B)**, stacked percentages for the five labels with the largest absolute library-mean composition differences. **(C)**, all finite library-pseudobulk gene effects, with follow-up genes overlaid; positive log2FC means IDD minus CTL and the dashed horizontal line marks q = 0.05. **(D)**, follow-up gene log2FC by cell-type pseudobulk; colours encode direction around zero. **(E)**, 12 follow-up gene effects from 8 CTL and 8 IDD libraries with descriptive intervals. **(F)**, TFRC, MST1, and SAA1 mean cell-level log1p (CP10 K) profiles across the 16 libraries. All inferential interpretation is anchored to libraries, not cells.

Figure 9A presents Spearman correlations among the 12 follow-up library means, without implying a mechanistic network. Figure 9B compares program scores by broad label. Figure 9C shows the follow-up effect directions on a compact bar scale, and Figure 9D reports program-level IDD-minus-CTL estimates with descriptive intervals. The observed correlation network in Figure 9E includes only library correlations with absolute rho≥0.45; the edges are an exploratory display of co-variation across 16 libraries. Figure 9F keeps MR log-odds, ferroptosis-panel membership, matrix presence, and scRNA log2FC in distinct evidence columns; no combined score or mediation path is inferred.

**FIGURE 9:**
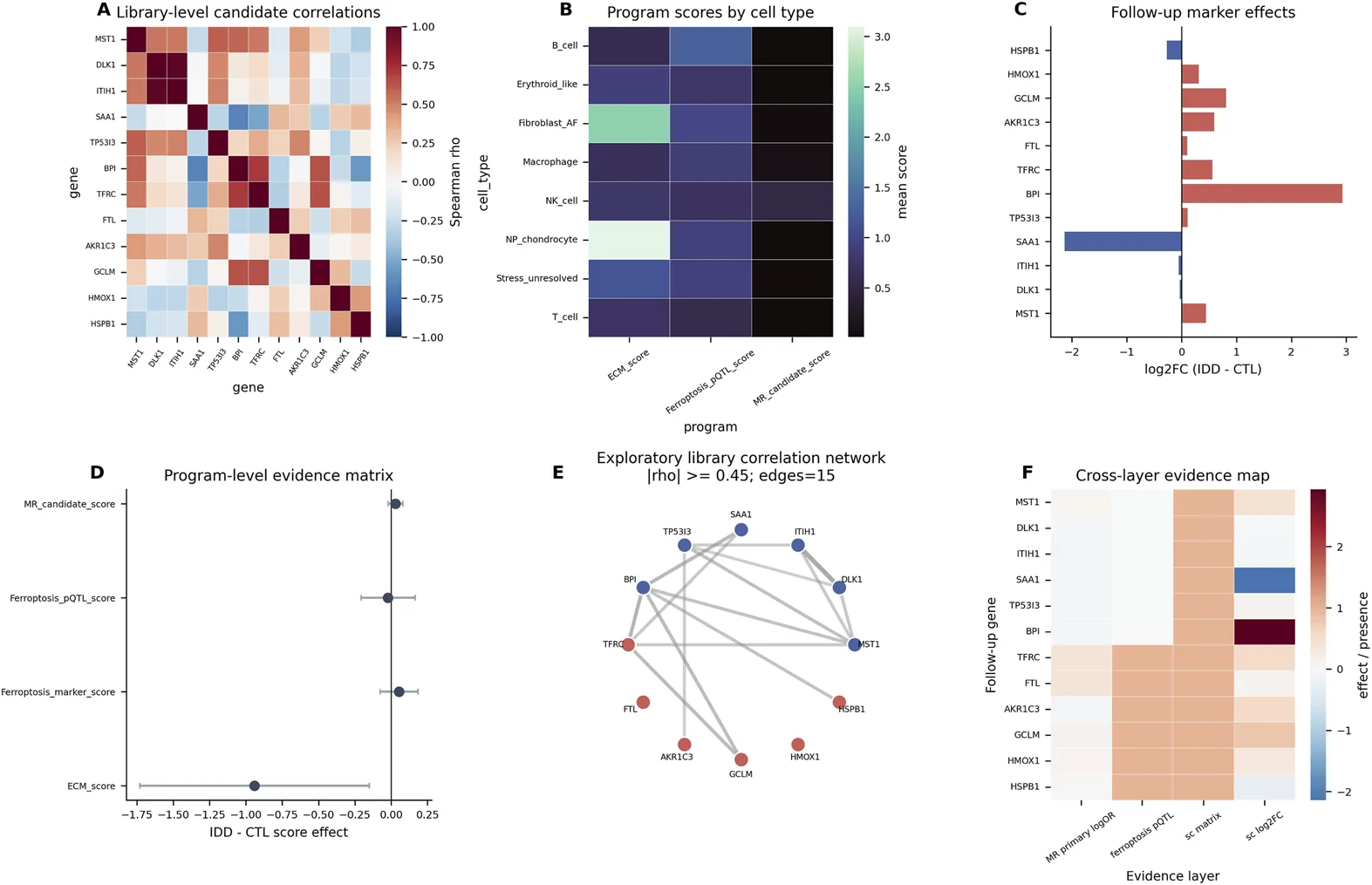
Cross-layer integration without a combined causal score. **(A)**, Spearman correlation among 12 follow-up gene library means. **(B)**, mean program scores by broad cell label. **(C)**, direction of the 12 follow-up library effects. **(D)**, IDD-minus-CTL program-score effects with descriptive intervals. **(E)**, exploratory observed correlation network showing only absolute rho≥0.45 edges across the 16 libraries; edges are not mechanistic claims. **(F)**, an evidence inventory with separate MR log-odds, ferroptosis-panel membership, matrix presence, and scRNA log2FC columns. Mixed estimands are not combined into one score.

### 3.5 Experimental qPCR validation

Figure 1 presents *in vitro* qPCR validation in a THP-1 macrophage-like inflammatory model. Relative to the control group, the inflammatory condition showed higher MST1 (1.34 ± 0.07), BPI (3.46 ± 0.28), and GCLM (1.70 ± 0.12) expression and lower SAA1 expression (0.31 ± 0.03), with control expression normalized to 1.00 (n = 4 independent biological replicates per group; all two-sided Welch P < 0.001). These expression directions were consistent with the library-level scRNA-seq effects, providing orthogonal experimental support for the selected candidates.

## 4 Discussion

This study integrates local pQTL-FinnGen screening with a library-aware single-cell atlas of human nucleus pulposus tissue. The genetic screen yielded four multi-SNP signals that passed Benjamini–Hochberg correction: MST1 showed an OR above 1, whereas DLK1, ITIH1, and SAA1 showed inverse directions. TP53I3 and BPI were retained only as nominal exploratory signals. In a separately prespecified ferroptosis-related panel, TFRC was the only gene with a BH-positive single-SNP screening result. The single-cell analysis then localized these candidates across eight broad cell populations and evaluated condition differences using the 16 GEO libraries rather than treating individual cells as independent replicates.

The genetic results should be interpreted as screening evidence rather than definitive causal protein effects. Although pQTL resources can connect genetically regulated protein abundance to disease phenotypes (Ferkingstad et al., 2021), valid MR interpretation depends on instrument relevance, independence, and exclusion-restriction assumptions (Lawlor et al., 2008; Skrivankova et al., 2021). In the present analysis, the exposure table did not contain a reference LD panel or an auditable clumping record. Consequently, the multi-SNP IVW, weighted-median, MR-Egger, heterogeneity, and leave-one-out results strengthen internal screening consistency but cannot establish that all variants represent independent instruments. This is why the manuscript consistently uses the terms local MR screening signal and genetically supported protein signal.

The directions of the four FDR-positive multi-SNP signals should also not be converted directly into gene-function claims. MST1 had a modest risk-direction estimate (OR 1.057), whereas DLK1, ITIH1, and SAA1 had ORs between 0.926 and 0.933. These estimates prioritize loci for replication, but the present data do not demonstrate the tissue source of the circulating protein signal, the relevant molecular isoform, or a mechanistic pathway within NP cells. The two nominal genes, TP53I3 and BPI, are particularly unsuitable for causal language because their BH q values exceeded 0.05.

TFRC provides a biologically coherent but still limited bridge to ferroptosis. Transferrin receptor 1 is central to cellular iron uptake and has been experimentally validated as a marker associated with ferroptotic states (Feng et al., 2020). Ferroptosis itself is defined by iron-dependent lipid peroxidation (Dixon et al., 2012), and prior IVDD studies have implicated iron homeostasis, HMOX1/GPX4 regulation, and ferroptosis-associated NP cell states (Lu et al., 2021; Yao et al., 2023; Zhang et al., 2021). In the present genetic screen, however, TFRC was supported by a single SNP. The OR of 1.471 and q = 0.023 therefore justify follow-up but not a claim that circulating TFRC is a confirmed causal driver of IVDD.

The scRNA-seq results emphasize heterogeneity rather than a uniform degenerative program. Eight broad labels were retained, including NP_chondrocyte, Fibroblast_AF, Macrophage, lymphoid populations, Erythroid_like, and Stress_unresolved. This broad cellular diversity is compatible with previous human single-cell studies showing multiple NP states and immune-cell involvement in disc degeneration (Zhang et al., 2021; Ling et al., 2022; Han et al., 2022). The macrophage component had the largest positive nominal IDD-minus-control composition difference (20.58 percentage points, P = 0.031), but its q value was 0.250. It is therefore an exploratory pattern rather than an FDR-supported compositional shift.

The same distinction applies to library-level expression effects. BPI showed the largest positive effect among the 12 displayed follow-up genes (log2FC 2.932, P = 0.035, q = 0.27), and GCLM was also nominally positive (log2FC 0.812, P = 0.044, q = 0.27); neither passed the within-follow-up BH threshold. Likewise, the Spearman correlation network summarizes co-variation across only 16 libraries and should not be interpreted as a regulatory or mechanistic network. Keeping MR log-odds, ferroptosis-panel membership, matrix presence, and scRNA log2FC in separate evidence columns avoids constructing a composite score from non-comparable estimands.

Figure 1 provides experimental qPCR validation using an ATCC THP-1 macrophage-like model and four representative genes spanning the genetic and single-cell layers (MST1, SAA1, BPI, and GCLM). The observed expression directions were consistent with the library-level scRNA-seq effects, supporting cross-platform concordance for these selected candidates. Because THP-1 cells are a macrophage-like model rather than primary nucleus pulposus cells, these findings should be confirmed in biologically appropriate disc models with prespecified stimulation conditions and primer sequences, verified amplification efficiency, raw Ct reporting, and independent biological replication. Future replication should follow established qPCR reporting standards and verify reference-gene stability (Bustin et al., 2009; Vandesompele et al., 2002).

Several features strengthen the analytical transparency of the study. Genetic screening was corrected across 1,723 gene-level primary tests, nominal exploratory genes were not promoted to FDR discoveries, and sensitivity analyses were presented within the limits of the available instruments. In the single-cell component, inferential comparisons were anchored to eight control and eight degenerated libraries, while UMAPs and feature maps were explicitly treated as descriptive. Low-specificity clusters were retained as Stress_unresolved rather than being forced into a canonical lineage, and ferroptosis-related expression follow-up was kept separate from primary MR significance.

Important limitations remain. The local pQTL analysis lacks an auditable LD-clumping framework and therefore requires replication with independently validated instruments. The FinnGen M13_INTERVERTEB endpoint represents other intervertebral disc disorders and is broader than a strictly radiographic IVDD phenotype. The genetic resources are predominantly European, limiting direct generalization across ancestries. The scRNA-seq dataset contains only 16 libraries, has an age imbalance between control and IDD groups, and provides a cross-sectional snapshot rather than longitudinal cell-state trajectories. Finally, nominal library-level findings and the THP-1 qPCR results require independent replication in primary or disc-relevant models before mechanistic or therapeutic conclusions are warranted. Figure 1 provides orthogonal experimental support, but the THP-1 macrophage-like system does not directly reproduce the cellular context of primary nucleus pulposus tissue.

## 5 Conclusion

This integrative analysis identifies a cautious set of genetically supported protein signals and maps selected candidates within the cellular context of human nucleus pulposus degeneration. MST1, DLK1, ITIH1, and SAA1 passed FDR correction in the multi-SNP local screen, while TFRC provided a separate BH-positive single-SNP ferroptosis-related signal. Library-aware scRNA-seq analysis revealed substantial cellular and between-library heterogeneity, with nominal composition and expression differences that did not survive within-panel FDR correction. The results therefore support candidate prioritization and cross-layer biological contextualization, not confirmed causal protein effects. Replication using LD-validated instruments, ancestry-diverse outcome data, independent single-cell cohorts, and additional experimental models will be required to establish mechanism and translational relevance. Figure 1 provides qPCR evidence that MST1, SAA1, BPI, and GCLM change in the same directions as the corresponding scRNA-seq effects in the THP-1 inflammatory model.

## Data Availability

The public datasets analyzed in this study can be found in the repositories identified in the article. The qPCR data generated in this study are available from the corresponding author upon reasonable request.
